# Shift work is associated with metabolic syndrome in male steel workers-the role of resistin and WBC count-related metabolic derangements

**DOI:** 10.1186/s13098-017-0283-4

**Published:** 2017-10-16

**Authors:** Yung-Chuan Lu, Chao-Ping Wang, Teng-Hung Yu, I-Ting Tsai, Wei-Chin Hung, I-Cheng Lu, Chia-Chang Hsu, Wei-Hua Tang, Jer-Yiing Houng, Fu-Mei Chung, Mei-Chu Yen Jean

**Affiliations:** 1 Division of Endocrinologic, E-Da Hospital, I-Shou University, Kaohsiung, 82445 Taiwan; 2 Division of Cardiology, E-Da Hospital, I-Shou University, Kaohsiung, 82445 Taiwan; 3 Division of Gastroenterology and Hepatology, Department of Internal Medicine, E-Da Hospital, I-Shou University, Kaohsiung, 82445 Taiwan; 4 Department of Emergency, E-Da Hospital, I-Shou University, Kaohsiung, 82445 Taiwan; 5 Department of Occupational Medicine, E-Da Hospital, I-Shou University, No. 1, Yi-Da Rd, Jiau-Shu Village, Yan-Chao Township, Kaohsiung, 82445 Taiwan; 6 Department of Nursing, I-Shou University, Kaohsiung, 82445 Taiwan; 7 School of Medicine for International Students, I-Shou University, Kaohsiung, 82445 Taiwan; 8 Department of Nutrition, Institute of Biotechnology and Chemical Engineering, I-Shou University, Kaohsiung, 82445 Taiwan; 9 Division of Cardiology, Department of Internal Medicine, National Yang-Ming University Hospital, Yilan, Taiwan

**Keywords:** Steel workers, Shift work, Metabolic syndrome, Body mass index, Fasting glucose, Triglyceride, High-density lipoprotein-cholesterol, Resistin, White blood cell count

## Abstract

**Aims:**

There is increasing evidence linking a shift work schedule with various adverse health effects. The present study aimed to examine the relationship between shift work and the metabolic syndrome (MetS) in male steel workers, and also the possible mechanism of shift work-related metabolic derangements.

**Methods:**

A total of 1732 men aged 42 ± 8 years were enrolled in this cross-sectional study, including 862 day workers and 870 shift workers. Circulating levels of resistin were measured by ELISA using monoclonal specific antibodies.

**Results:**

The shift workers had higher rates of MetS and its components (central obesity, hypertension, and hypertriglyceridemia) than the day workers. In multiple logistic regression analysis, shift work was independently associated with MetS. In further analysis, the shift workers had elevated circulating levels of resistin (13 ± 10 vs. 10 ± 7 ng/mL) and total white blood cell (WBC) count (6.865 ± 1.819 vs. 6.304 ± 1.547 10^9^/L) than the day workers. In addition, both resistin level and total WBC count were significantly associated with shift work, MetS, and its components (body mass index, fasting glucose, triglyceride, and high-density lipoprotein-cholesterol levels), and plasma resistin levels were significantly associated with total WBC count (β = 0.34, p < 0.0001).

**Conclusion:**

Shift work was independently associated with MetS in male steel workers. Resistin and WBC count were associated with shift work-related metabolic derangements.

## Background

Shift work is defined that takes place on a schedule outside the traditional 9 a.m.–5 p.m. day. It can involve evening or night shifts, early morning shifts, and rotating shifts [1]. Shift work is increasing worldwide, and as many as 20% of workers in industrialized nations are shift workers [2]. Shift work has been reported to disrupt the circadian rhythm, sleep and work-life balance, and to increase the risk of metabolic syndrome (MetS) [3], coronary heart disease [4], mental and behavioral disorders [5], and sleep disturbances [6]. The major function of the circadian system is internal cycling of physiological and metabolic events [7]. Many physiological processes display a day-night rhythms, and feeding behaviour, lipid and carbohydrate metabolism and blood pressure are subject to daily variations.

The prevalence of MetS is increasing globally, including Taiwan. The components of MetS including dyslipidemia, dysglycemia, high blood pressure, and being overweight/obese tend to cluster together, and they are thought to be related to lifestyle habits including physical activity, nutrient intake, alcohol consumption, and smoking [8–11]. Few studies have examined the relationships between shift work and MetS taking into consideration the various factors associated with shift work that might lead to MetS. In addition, shift work may be responsible for increased body fatness and inflammatory markers, higher blood pressure levels, and chronic low-grade inflammation to play an important role in the development of MetS [3, 12–16].

The detrimental effects of shift work on MetS and the induction of inflammatory cytokines and associated factors raise the possibility that shift work may increase the risk of MetS. To clarify this hypothesis, we investigated the association between shift work and features of National Cholesterol Education Program Adult Treatment Panel III (NCEP-ATP III)-defined MetS [17]. The possible mechanisms by which shift work affects MetS were also investigated by measuring inflammatory markers including resistin and peripheral white blood cell (WBC) count in a large population-based sample of male Chinese steel workers.

## Subjects and methods

### Study design and participants

This study was conducted at a hospital from January 1 to December 31, 2016. Workers at two steel plants in southern Taiwan who underwent annual health examinations were included in this cross-sectional survey study. In total, 1732 men (862 day workers, 870 backward rotating shift workers) of self-reported Chinese ancestry aged 42 ± 8 years were selected as the study cohort, after 69 had been excluded due to a history of acute or chronic inflammatory disease, allergic disease, autoimmune disease, or cancer. The work schedule was classified as daytime work (8:00–17:00) and shift work, which included rotating shifts of night (23:00–7:00), afternoon (15:00–23:00), and morning (7:00–15:00) shifts. None of the subjects changed schedule during the study period, and they were all engaged in this schedule at baseline. This study was approved by the Human Research Ethics Committee of Kaohsiung E-Da Hospital, I-Shou University. Written informed consent was obtained from all participants.

### Data collection and measures

Self-administered questionnaires were distributed and collected on the day of the health examination. Information on basic demographic characteristics and lifestyle, such as age, sex, job type, sleep quality, health condition, physical exercise, smoking habit, and alcohol consumption were ascertained using the questionnaire. Physical exercise was assessed by the question “How often did you exercise during the past month?” The response options were: hardly ever, once, and twice or more. For working schedule, the participants were asked whether they were daytime workers (8:00–17:00) or shift workers working rotating shifts of morning (7:00–15:00), afternoon (15:00–23:00), and night (23:00–7:00) shifts. Sleep quality was assessed by the question “How often did you have poor sleep during the past month?” The response options were: almost never, sometimes, and often or almost always.

The smoking status of the subjects was classified as never having smoked, former smoker (quit smoking for at least 1 year), or current smoker. Alcohol drinking and betel quid chewing status were classified as never drink or chew betel quid, former drinker or betel quid chewer (quit drinking or betel quid chewing for at least 1 year), or current drinker or betel quid chewer. In this study, former and current drinkers and betel quid chewers were analyzed as a single group [18]. In addition, body mass index (BMI) was calculated as weight (in kilograms) divided by height (in meters) squared. MetS was defined according to the NCEP-ATP III with a modified definition of central obesity [17]. MetS was diagnosed when a subject met three or more of the following criteria: (1) arterial blood pressure ≥ 130/85 mmHg, (2) central obesity (waist circumference, males ≥ 90 cm; females ≥ 80 cm), (3) serum triglyceride level ≥ 150 mg/dL, (4) serum high-density lipoprotein cholesterol (HDL-C) < 40 mg/dL in males or < 50 mg/dL in females; and (5) fasting plasma glucose concentration ≥ 100 mg/dL or a previous diagnosis of type 2 diabetes.

### Laboratory measurements

Peripheral blood samples were taken from the antecubital vein of the workers after fasting for at least 8 h. Complete blood cell count and levels of serum glucose, HbA1c, uric acid, and lipid profiles [including plasma triglycerides, total cholesterol, low-density lipoprotein cholesterol (LDL-C), and HDL-C] were also measured during the health checkups and were determined in all workers using standard commercial methods with a parallel, multichannel analyzer (Hitachi 7170A, Tokyo, Japan) as described in our previous report [19, 20]. Peripheral leukocyte analysis was performed using an automated cell counter (XE-2100 Hematology Alpha Transportation System, Sysmex Corporation, Kobe, Japan). The concentrations of plasma resistin were determined using commercial enzyme immunoassay kits (Phoenix Pharmaceuticals, Belmont, CA). The intraassay coefficient of variation was 2.1–5.2% for resistin. Samples were measured in duplicate in a single experiment.

### Statistical analysis

The data are presented as the mean ± SD. All statistical analyses were performed using SAS software (version 8.0; SAS Institute, Cary, NC). Statistical differences between variables were compared using unpaired Student’s *t*-tests for normally distributed variables. Categorical variables were recorded as frequencies and/or percentages, and intergroup comparisons were analyzed using the Chi square test. Multiple logistic regression analysis was used to assess independent associations between the variables of interest and the presence of MetS. In addition, simple and multiple linear regression analyses were used to examine associations between peripheral total WBC count and resistin and the values of other parameters. All statistical analyses were two-sided, and a p value < 0.05 was considered to be statistically significant.

## Results

The clinical characteristics of the participants are presented in Table 1. Of the 1732 participants, 870 (50.2%) had been engaged in shift work for at least 1 year. Compared to the daytime workers, significantly more shift workers were current smokers, former and current betel quid users, hardly ever engaged in physical exercise, and sometimes and often or almost always had poor sleep (all p < 0.05). They also had higher rates of central obesity (p = 0.03), hypertension (p = 0.0002), hyper-triglyceridemia (p = 0.04), and MetS (p = 0.03) than the daytime workers. In addition, the shift workers had higher systolic blood pressure (SBP), diastolic blood pressure (DBP), BMI, waist circumference, total WBC count, and higher levels of fasting glucose, triglycerides, uric acid, and resistin than the daytime workers. Moreover, the shift workers had a lower level of HDL-C than the daytime workers (Table 2). There were no significant differences in age, HbA1C, total cholesterol, LDL-C level, percentage of participants aged 25–40 and 50–60 years, alcohol consumption, former smoking, engaging in physical exercise once per month, low HDL-C, and dysglycemia between the two groups.

**Table 1:** Clinical characteristics of the study subjects

Parameter	Shift work	Day work	p value
No	870	862	
Age (years) (n, %)			
25–40	338 (38.9)	354 (41.1)	0.35
40–50	411 (47.2)	365 (42.3)	0.04
50–60	113 (13.0)	122 (14.2)	0.48
> 60	8 (0.9)	21 (2.4)	0.02
Alcohol use (n, %)			
Never	470 (54.0)	501 (58.1)	0.09
Former	25 (2.9)	20 (2.3)	0.47
Current	375 (43.1)	341 (39.6)	0.13
Smoking (n, %)			
Never	404 (46.4)	506 (58.7)	< 0.0001
Former	104 (12.0)	101 (11.7)	0.88
Current	362 (41.6)	255 (29.6)	< 0.0001
Betel quid use (n, %)			
Never	636 (73.1)	747 (86.7)	< 0.0001
Former	94 (10.8)	43 (5.0)	< 0.0001
Current	140 (16.1)	72 (8.4)	< 0.0001
Physical exercise in the past month (n, %)			
Hardly ever	225 (25.9)	127 (14.7)	< 0.0001
Once	86 (9.9)	87 (10.1)	0.91
Twice or more	559 (64.3)	648 (75.2)	< 0.0001
Poor sleep (n, %)			
Almost never	600 (69.0)	683 (79.2)	< 0.0001
Sometimes	181 (20.8)	122 (14.2)	0.002
Often or almost always	89 (10.2)	57 (6.6)	0.02
Central obesity (n, %)	338 (38.9)	292 (33.9)	0.03
Hypertension (n, %)	436 (50.1)	353 (41.1)	0.0002
Hypertriglyceridemia (n, %)	315 (36.2)	271 (31.4)	0.04
Low–high-density lipoprotein-cholesterol (n, %)	224 (25.8)	224 (26.0)	0.91
Dysglycemia (n, %)	300 (34.5)	288 (33.4)	0.64
Metabolic syndrome (n, %)	276 (31.7)	233 (27.0)	0.03

**Table 2:** Biochemical characteristics of the study subjects

Parameter	Shift work	Day work	p value
No	870	862	
Age (years, mean ± SD)	42 ± 8	43 ± 8	0.79
Systolic blood pressure (mmHg)	130 ± 16	126 ± 15	< 0.0001
Diastolic blood pressure (mmHg)	84 ± 12	81 ± 11	< 0.0001
Body mass index (kg/m^2^)	26.0 ± 4.0	25.6 ± 3.8	0.03
Waist circumference (cm)	88.1 ± 10.1	81.5 ± 10.6	< 0.0001
Fasting glucose (mmol/L)	5.4 ± 0.8	5.2 ± 0.4	0.02
HbA1C (%)	5.6 ± 0.7	5.5 ± 0.7	0.31
Total cholesterol (mmol/L)	5.1 ± 1.1	5.0 ± 0.9	0.30
Triglyceride (mmol/L)	1.7 ± 1.1	1.3 ± 0.9	0.002
HDL-cholesterol (mmol/L)	1.2 ± 0.3	1.3 ± 0.4	0.002
LDL-cholesterol (mmol/L)	2.9 ± 0.9	2.7 ± 0.8	0.19
Uric acid (μmol/L)	392.6 ± 77.3	362.8 ± 89.2	0.02
Total WBC count (10^9^/L)	6.865 ± 1.819	6.304 ± 1.547	< 0.0001
Resistin (ng/mL)^a^	13 ± 10	10 ± 7	0.02

Data are mean ± SD

*HDL* high-density lipoprotein, *LDL* low-density lipoprotein, *WBC* white blood cell. p values were calculated using the independent sample *t* test for numerical data

^a^Resistin levels were measured in 107 shift workers and 100 day workers

**Fig. 1:**
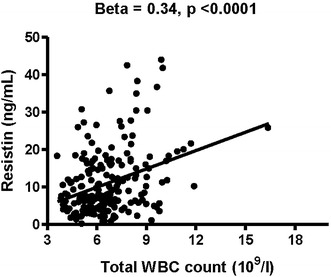
Association between plasma resistin concentration and total white blood cell (WBC) count. Plasma resistin concentration was significantly positively associated with total WBC count

Multivariate logistic regression analysis showed that shift work had an odds ratio of 2.26 for the risk of MetS (p = 0.02, Table 3). In age-adjusted association analysis including inflammatory markers and risk factors for MetS, resistin was significantly positively associated with BMI (β = 0.14, p = 0.048), fasting glucose (β = 0.24, p = 0.001), HbA1C (β = 0.15, p = 0.04), triglycerides (β = 0.21, p = 0.01), shift work (β = 0.17, p = 0.02), and MetS (β = 0.17, p = 0.02), while total WBC count was significantly positively associated with BMI (β = 0.25, p < 0.0001), waist circumference (β = 0.26, p < 0.0001), SBP (β = 0.16, p < 0.0001), DBP (β = 0.15, p < 0.0001), fasting glucose (β = 0.09, p < 0.0001), HbA1C (β = 0.17, p < 0.0001), triglycerides (β = 0.25, p < 0.0001), uric acid (β = 0.10, p < 0.0001), shift work (β = 0.16, p < 0.0001), and MetS (β = 0.22, p < 0.0001). In addition, HDL-C was significantly negatively associated with both resistin (β = −0.23, p = 0.001) and total WBC count (β = −0.20, p < 0.0001) (Table 4). Furthermore, resistin was significantly associated with total WBC count (β = 0.34, p < 0.0001) (Fig. 1).

**Table 3:** Multiple logistic regression analysis with the presence of metabolic syndrome as the dependent variable

	Exp(B)	95% confidence interval	p value
Age	1.03	0.98–1.09	0.22
Alcohol use	0.33	0.07–1.60	0.17
Smoking	1.07	0.50–2.30	0.86
Betel quid use	2.04	0.90–4.64	0.09
Poor physical exercise	1.05	0.45–2.44	0.91
Poor sleep	0.94	0.37–2.38	0.89
Shift work	2.26	1.13–4.51	0.02

**Table 4:** Association of covariates with total WBC count and resistin

Factor	Total WBC count		Resistin	
	β-coefficient (95% CI)^a^	p value	β-coefficient (95% CI)^a^	p value
Body mass index	0.25 (0.09 to 0.13)	< 0.0001	0.14 (0.00 to 0.62)	0.048
Waist circumference	0.26 (0.04 to 0.05)	< 0.0001	0.12 (− 0.02 to 0.21)	0.10
Systolic BP	0.16 (0.01 to 0.02)	< 0.0001	0.13 (− 0.01 to 0.16)	0.08
Diastolic BP	0.15 (0.02 to 0.03)	< 0.0001	0.11 (− 0.03 to 0.21)	0.14
Fasting glucose	0.09 (0.00 to 0.01)	< 0.0001	0.24 (0.07 to 0.28)	0.001
HbA1C	0.17 (0.23 to 0.41)	< 0.0001	0.15 (0.05 to 3.54)	0.04
Total cholesterol	0.04 (0.00 to 0.01)	0.12	− 0.01 (− 0.04 to 0.03)	0.90
Triglyceride	0.25 (0.00 to 0.01)	< 0.0001	0.21 (0.01 to 0.03)	0.01
HDL-cholesterol	− 0.20 (− 0.04 to − 0.03)	< 0.0001	− 0.23 (− 0.26 to − 0.06)	0.001
LDL-cholesterol	0.03 (− 0.00 to 0.01)	0.18	− 0.04 (− 0.05 to 0.03)	0.57
Uric acid	0.10 (0.07 to 0.19)	< 0.0001	0.11 (− 0.19 to 1.55)	0.12
Shift work	0.16 (0.39 to 0.70)	< 0.0001	0.17 (0.49 to 5.38)	0.02
Metabolic syndrome (NCEP-ATP III)^b^	0.22 (0.64 to 0.99)	< 0.0001	0.17 (0.53 to 5.73)	0.02

*WBC* white blood cell, *BP* blood pressure, *HDL* high-density lipoprotein, *LDL* low-density lipoprotein

^a^Adjusted for age using multiple linear regression analysis

^b^The definition and criteria of NCEP-ATP III metabolic syndrome are described in the text

## Discussion

Our results showed that shift work was significantly associated with MetS. Furthermore, resistin and WBC count may be involved in the pathogenesis of shift work-associated metabolic derangements. To our best of knowledge, this study is the first to show an association between shift work and MetS, and that chronic inflammation induced by shift work may lead to metabolic dysfunction.

Our findings regarding shift work and MetS are consistent with those of previous studies [21–25]. A higher risk of having more MetS components has been reported in a range of occupations including road builders, police officers, nurses, and factory workers in different countries and areas [21, 22, 24–26]. The observed increase in the risk of MetS in shift workers may be explained by several mechanisms. First, a theoretical review showed that sleep and circadian disruption in humans alters the gut microbiota, thereby contributing to an inflammatory state and metabolic disease associated with shift work [27]. Furthermore, shift workers have been reported to gain weight, experience disruption of the circadian rhythm, and impairment of sleep that results in a higher risk of MetS [3, 6, 28–30]. Previous study has also reported associations between night or shift work and increased food intake, a preference for carbohydrate-rich foods, and alterations in lipid parameters, especially triglyceride levels [31]. Moreover, adverse cardiometabolic effects of circadian misalignment produced by shifting eating and sleeping times 12 h out of phase from habitual times have been shown to result in increased levels of glucose, despite increased levels of insulin, decreased leptin, a completely reversed daily cortisol rhythm, increased mean arterial pressure, and reduced sleep efficiency [32].

Numerous epidemiological and clinical studies have shown that leukocytosis is an independent predictor of insulin resistance, type 2 diabetes, and cardiovascular diseases [33–35]. The elevated peripheral WBC count in the shift workers in the present study may suggest a mechanism for the pathogenesis of shift work-related MetS. The WBC in shift workers may be activated by reactive oxygen species or adipocytokines [36]. Activated leukocytes release many kinds of cytokines, including tumor necrosis factor-α, nuclear transcription factor-kappaB, and interleukin, superoxide radicals, and proteases, all of which contribute to insulin resistance, MetS, and atherosclerosis [37]. In addition, we also found that plasma resistin levels were higher in the shift workers, and that they were significantly associated with total leukocyte count. We speculate that resistin may enhance the release and activation of leukocytes, and that this is probably the main source of resistin at the site of inflammation which then contributes to the development and progression of insulin resistance and MetS.

Resistin is a circulating protein of 114 amino acids which belongs to the resistin-like family [38]. Resistin is expressed in macrophages and plays an important role in inflammation throughout the body [39]. Several studies from the 2002 to 2008 have also shown that resistin is regulated by insulin, glucose, growth hormone, and thiazolidinediones [40–43]. Rodent studies have suggested that resistin protein is a link between obesity, insulin resistance, and diabetes [44–46]. In addition, Palanivel et al. [47] reported that mice injected with recombinant resistin or overexpressing resistin protein had impaired glucose tolerance and insulin action. Furthermore, Sheng et al. [48] reported that human hepatic cells overexpressing resistin had impaired glucose uptake and glycogen synthesis. Because of its link with obesity, inflammation, and insulin resistance, resistin has been tagged as a potential marker of MetS. In the present study, we found that plasma levels of resistin were significantly increased in the shift workers, suggesting that resistin synthesis or secretion was enhanced. Resistin is a well-known mediator of chronic inflammation that contributes to the pathogenesis of insulin resistance, dyslipidemia, hypertension, MetS, and atherosclerosis [44–46, 49, 50]. Taken together, it is plausible that shift work may induce MetS through a low-grade chronic inflammatory process.

## Limitations

There are several limitations to the present study. First, this was a cross-sectional study and thus it was difficult to investigate the causal relationship between shift work and inflammatory markers. Second, the absence of adequate data for nutrition to assess the daily energy and nutrient intake of the study subjects may have resulted in some bias. Third, we did not consider other confounders that may contribute to the shift work-related development of MetS such as psychosocial stress including income, job, and behavioral stress, and occupational characteristics including tenure, work intensity, and hazardous materials handled at work. Fourth, although we focused on manual workers, a more homogenous population should be selected for future studies as each of our participants had different work tasks, environment, and exposure to hazardous materials, as well as different work schedules according to their education level. Fifth, we did not establish a detailed definition of former shift workers; thus, additional studies may be required to confirm differences in inflammatory markers between past shift workers and recent former shift workers and the reversibility of shift work-induced adverse health effects.

## Conclusion

Our results indicate that shift work is independently associated with a higher prevalence of MetS, and that is has a detrimental influence on central obesity, hypertension, and hyper-triglyceridemia. Chronic inflammation may contribute to shift work-related metabolic derangements. The results of our study raise an important epidemiological issue with regards to MetS, and further large-scale cohort studies are warranted to validate our findings.

## Abbreviations

MetS: metabolic syndrome
NCEP-ATP III: National Cholesterol Education Program Adult Treatment Panel III
WBC: white blood cell
HDL-C: high-density lipoprotein cholesterol
LDL-C: low-density lipoprotein cholesterol
SBP: systolic blood pressure
DBP: diastolic blood pressure
BMI: body mass index
